# Characteristics of Children With Kawasaki Disease-Like Signs in COVID-19 Pandemic: A Systematic Review

**DOI:** 10.3389/fped.2021.625377

**Published:** 2021-03-18

**Authors:** Parham Mardi, Marzieh Esmaeili, Parisa Iravani, Mohammad Esmail Abdar, Kumars Pourrostami, Mostafa Qorbani

**Affiliations:** ^1^Student Research Committee, Alborz University of Medical Sciences, Karaj, Iran; ^2^Department of Health Information Management, School of Allied Medical Sciences, Tehran University of Medical Sciences, Tehran, Iran; ^3^Pediatrics Department, Isfahan University of Medical Sciences, Isfahan, Iran; ^4^Department of Pediatrics, Child Growth and Development Research Center, Research Institute for Primordial Prevention of Non-communicable Disease, Isfahan University of Medical Sciences, Isfahan, Iran; ^5^Social Determinants of Health Research Center, Alborz University of Medical Sciences, Karaj, Iran; ^6^Dietary Supplements and Probiotic Research Center, Alborz University of Medical Sciences, Karaj, Iran; ^7^Non-communicable Diseases Research Center, Alborz University of Medical Sciences, Karaj, Iran; ^8^Chronic Diseases Research Center, Endocrinology and Metabolism Population Sciences Institute, Tehran University of Medical Sciences, Tehran, Iran

**Keywords:** COVID-19, Kawasaki, MIS-C, hyperinflammatory, children

## Abstract

Recent studies have shown that several children diagnosed with COVID-19 have developed Kawasaki Disease (KD)-like symptoms. This systematic review aims to assess the demographic, laboratory, and clinical characteristics of children with KD-like syndrome during the COVID-19 pandemic and evaluate efficacy of treatments and patients' outcome. A comprehensive search was carried out systematically through PubMed, Scopus, and Web of Science (WoS), medRxiv, and bioRxiv by two reviewers independently for all studies or preprints data on the demographic, laboratory, and clinical characteristics of children with K.D-like signs during the COVID-19 outbreak. Overall, 378 studies were identified by the systematic search, of which 25 studies were included in the study. The included studies involved 599 patients in total. Thirteen studies (52%) were case reports or case series, and the rest (48%) were cohort studies. In 19 studies, patients were diagnosed with Multisystem inflammatory syndrome in children (MIS-C). In 16 studies COVID-19 was diagnosed in all patients based on their polymerase chain reaction result, serological findings, and computed tomography results. Higher C-reactive protein and erythrocyte sedimentation rate level were the most prevalent laboratory findings. In most studies, patients had leucopenia with marked lymphopenia, hypoalbuminemia, and increased ferritin, as well as hyponatremia. Abnormal echocardiography and respiratory outcomes were the most common clinical outcomes. In 11 studies, all patients required intensive care unit admission. Findings of the present systematic review show that the incidence of KD-like syndrome in the COVID-19 pandemic increased significantly. Moreover, this study offers new insights in the KD-like syndrome pathogenesis and clinical spectrum during COVID-19 pandemic.

## Background

Coronavirus Disease 2019 (COVID-19), caused by SARS-CoV-2, has created a global pandemic. Millions of people have been infected, and thousands have lost their lives (1). The main clinical manifestations of this disease are fever, coughing, shortness of breath, fatigue, and malaise (2).

At the beginning of the crisis, children were rarely reported as infected. These reports led to the assumption that children are immune to this virus. With the continuation of the pandemic and the quarantines that caused many families to stay close in their houses, more children with COVID-19 were identified (3). The children infected with the novel coronavirus can be asymptomatic or present with fever, dry coughs, fatigue, and a few upper respiratory symptoms, including nasal congestion and runny nose (4, 5). Even though the disease is not as severe as in adults, yet some case reports and case series have reported more severe symptoms in some cases, compared to the other symptomatic children (6).

Kawasaki disease (KD) is one of the vasculitis of medium-sized vessels, that only affects children. Although no identified etiology can clearly explain this disease, some assumptions consider viral infections such as coronavirus family act as a trigger in genetically predisposed children (7, 8).

Recent studies have shown that several children diagnosed with COVID-19 have developed symptoms such as prolonged fever, bilateral conjunctival injections, changes in the lips and oral cavity, cervical lymphadenopathy, extremity changes, and polymorphous rash that are similar to those of KD or Hyper inflammatory syndrome (9, 10). On the other hand, there are some differences between KD-like syndrome and KD including a faster advancement of symptoms, especially fever and multisystem organ dysfunction such as cardiac and respiratory dysfunction along with a current or recent COVID-19 diagnosis in KD-like syndrome patients. Also, KD-like syndrome patients suffer a tachycardia and are at higher risk of cardiac or respiratory arrest. Moreover, Primary studies pointed out that KD-like syndrome is associated with a poorer prognosis for the patients (8, 11). On the ground of lack of information on KD-like syndrome, the objective of this study is to assess the demographic, laboratory, and clinical characteristics of children with KD-like syndrome during the COVID-19 pandemic and evaluate efficacy of treatments and patients' outcome.

## Materials and Methods

This study is reported based on the Preferred Reporting Items for Systematic Reviews and Meta-Analyses (PRISMA) guidelines (12, 13).

### Eligibility Criteria

The following inclusion criteria were used: (I) Published in the English language; (II) Full-text available; (III) observational studies (including case reports and case series); (IV) studies reporting the characteristics and outcome of children with KD-like syndrome during the COVID-19 outbreak; (V) patients in the study fulfill the classical Kawasaki criteria according to the American Heart Association indications (2017) (fever for ≥5 days plus four or more clinical criteria, including bilateral bulbar non-exudative conjunctivitis, changes of the lips or oral cavity, non-suppurative laterocervical lymphadenopathy, polymorphic rash, erythema of the palms and soles, firm induration of the hands or feet, or both) or KD-like syndrome criteria, based on CDC recommendation [An individual under 21 years presenting with fever, laboratory evidence of inflammation, and evidence of clinically severe illness requiring hospitalization, with multi-system (two or more) organ involvement (cardiac, renal, respiratory, hematologic, gastrointestinal, dermatologic, or neurological); AND No alternative plausible diagnoses; AND Positive for current or recent SARS-CoV-2 (COVID-19) infection by reverse-transcriptase polymerase chain reaction (RT-PCR), serology or antigen test; or COVID-19 exposure within the 4 weeks prior to the onset of symptoms].

### Information Sources

A comprehensive search was carried out systematically through PubMed, Scopus, and Web of Science (WoS), databases (from their inception until July 1, 2020), and medRxiv, bioRxiv (between January 1, 2020, and July 12, 2020) by two reviewers independently.

### Search Strategy

A search study was designed comprising of three concepts, the 2019 novel coronavirus disease, KD disease, and child. The following algorithm was used for screening the title and abstract (Supplementary Table 1); (“covid 19” OR “covid-19” OR “^*^covid-19^*^” OR “^*^covid^*^” OR “^*^SARS-CoV-2^*^” OR “^*^2019-nCoV^*^” OR “^*^novel coronavirus^*^” OR “^*^new coronavirus^*^” OR “^*^coronavirus^*^”) AND (Kawasaki OR KD OR Kawasaki-like OR multi-system OR multi-system) AND inflammatory AND (syndrome^*^ OR disease) OR MISC OR MIS-COR PIMS OR [inflammatory AND (multi-system OR multi-system) AND (syndrome^*^ OR disease^*^) OR Systemic Inflammatory Response Syndrome OR hyperinflammatory] AND (child^*^ OR pediatric).

### Study Selection

The study selection process was done in multiple phases via the EndNote reference management software to manage the acquired articles. At first, duplicate articles were identified through the software and manually. Then, in the screening phase, the title and abstract of the studies were examined based on the including criteria. Afterward, the full texts were screened in detail if needed. The selection process was done by two authors independently (M.E. & M.Q.). They came to an agreement regarding the conflicting results.

### Data Collection Process and Data Items

Two independent researchers filled data extraction forms containing age, gender, journal, study type, sample size, clinical characteristics, laboratory findings, and outcomes. Another researcher resolved conflicts.

### Quality Assessment

Quality assessment (QA) of case-reports/case-series studies was assessed using the case-report (CARE) 13-item guideline (14). This guideline comprises 30 sub-items. Each item ratings are yes, or no, and the final QA score was the sum of sub-items. For QA of cohort studies, the Newcastle-Ottawa scale was used (maximum: nine stars) (15). This tool consists of three domains; comparability (maximum: two stars), selection (maximum: four stars), and outcome (maximum: three stars). The QA was carried out independently by two investigators (MQ and PM) addressing the items reported in the guidelines.

## Results

### Study Selection

**Figure 1:**
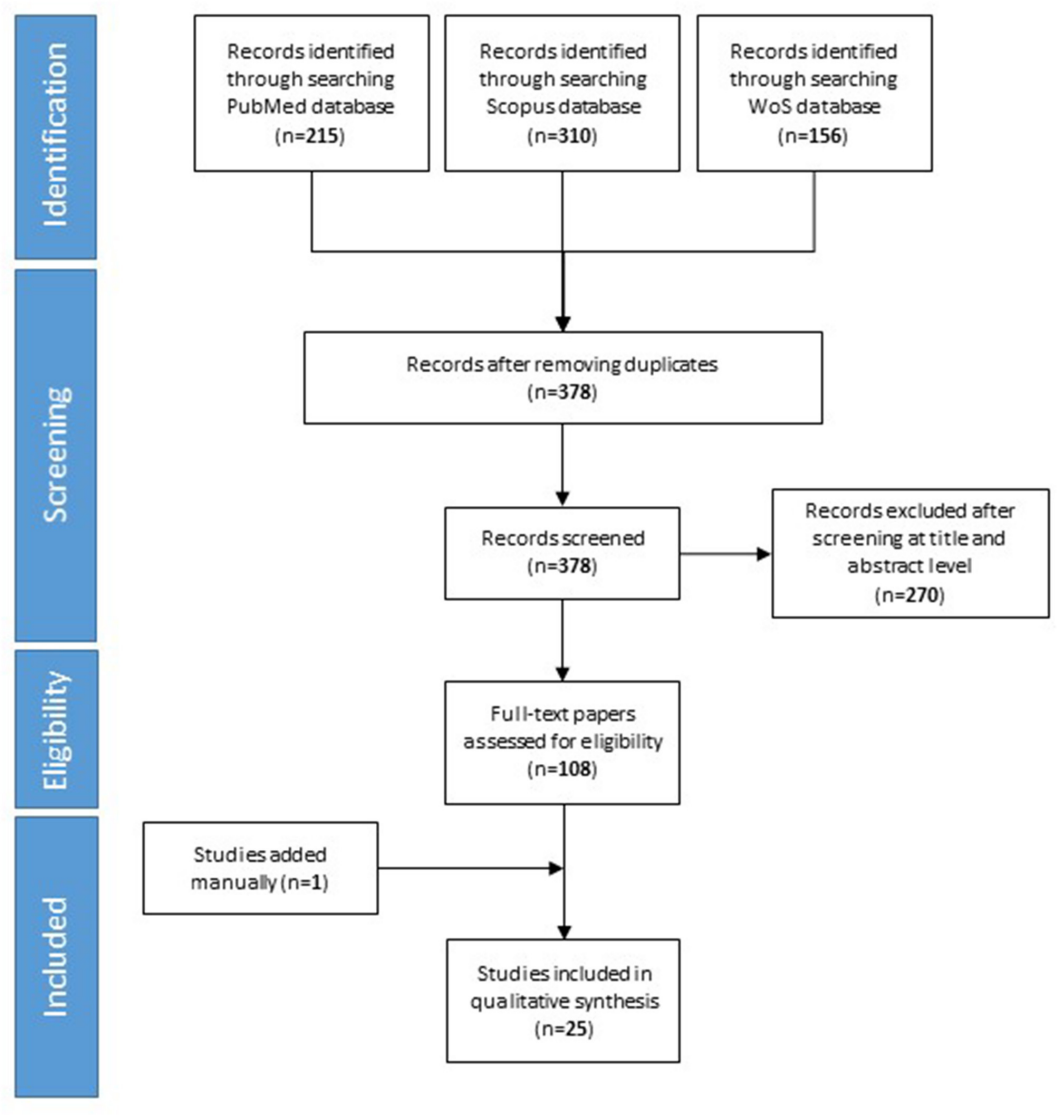
PRISMA diagram for searching resources.

Our searches yielded 215 studies from PubMed, 310 studies from Scopus, and 156 study from Web of science. After removal of duplicates, we assess 378 studies for eligibility. We excluded 270 studies based on their title and abstract. Finally, 25 studies included in the qualitative synthesis. The detailed flow diagram is shown in Figure 1.

### Study Characteristics

Table 1 shows the characteristics of the 25 included studies. Ten were conducted in the USA, 12 in Europe, 2 in India and 1 in Brazil. The included studies involved 599 patients in total; 347 (57.92%) were males. Thirteen studies (52%) were case reports or case series, and the rest (48%) were cohort studies. The minimum and maximum age for the patients ranged from 6 months to 16.6 years in the individual studies, respectively. In 19 studies, patients were diagnosed based on the multi-system inflammatory syndrome in children criteria (MIS-C) (KD-like syndrome) and in 6 studies patients fulfilled Kawasaki disease criteria.

**Table 1:** General characteristics of included studies.

**References**	**Date**	**Population**	**Country**	**Study type**	**Sample size**	**Gender (male/female)**	**Age/Median (range)**	**Quality score**
Jones et al. (16)	N.R.	Diagnosed with K.D.^*^	USA	Case report	1	0/1	0.5	93%^a^
Grimaud et al. (17)	15–27 April, 2020	MIS-C^**^	France	Retrospective cohort	20	10/10	10 (2.9–15)	7^b^
Toubiana et al. (18)	Apr 27–May 11, 2020	MIS-C^**^	France	Prospective cohort	21	9/12	7.9 (3.7–16.6)	8^b^
Rivera-Figueroa et al. (19)	NR	MIS-C^**^	USA	Case report	1	1/0	5	85%^a^
Balasubramanian et al. (20)	NR	MIS-C^**^	India	Case report	1	1/0	8	81%^a^
Verdoni et al. (21)	Feb 18–Apr 20, 2020	Diagnosed with K.D.^*^	Italy	Retrospective cohort	10	7/3	7.5 (2.9–16.0)	8^b^
Belhadjer et al. (22)	Mar 22–Apr 30, 2020	MIS-C^**^	France and Switzerland	Case series	35	18/17	10 (2–16)	78%^a^
Licciardi et al. (23)	14 and 18 April, 2020	MIS-C^**^	Italy	Case report	2	2/0	7 and 12	85%^a^
Deza Leon et al. (24)	NR	MIS-C^**^	USA	Case report	1	0/1	6	78%^a^
Dolinger et al. (25)	NR	MIS-C^**^	USA	Case report	1	1/0	14	81%^a^
Riphagen et al. (26)^***^	mid-April, 2020	MIS-C^**^	UK	Case series	8	5/3	8 (4–14)	74%^a^
Waltuch et al. (27)	April, 2020	MIS-C^**^	USA	Case series	4	3/1	11 (5–13)	81%^a^
Labé et al. (28)	N.R.	Diagnosed with K.D.^*^	France	Case report	1	1/0	3 and 6	93%^a^
Toubiana et al. (29)	Apr 27–May 7, 2020	Diagnosed with K.D.^*^	France	Retrospective cohort	17	7/10	7.5 (3.7–16.6)	7^b^
Chiotos et al. (30)	May, 2020	MIS-C^**^	USA	Case series	6	1/5	7.5 (5–14)	74%^a^
Rauf et al. (31)	Late April, 2020	MIS-C^**^	India	Case report	1	1/0	5	93%^a^
Pouletty et al. (32)	April, 2020	Diagnosed with K.D.^*^	France	Cohort	16	8/8	10 (4.7–12.5)	8
Dufort et al. (33)	March 1– May 10, 2020	MIS-C^**^	USA	Cohort	99	53/46	NR	8
Capone et al. (34)	April 17—May 13, 2020	MIS-C^**^	USA	Cohort	33	13/20	8.6 (5.5–12.6)	7
Whittaker et al. (35)	March 23– May 16, 2020	MIS-C^**^	UK	Cohort	58	38/20	9 (5.7–14)	8
Dallan et al. (36)	April 2020	MIS-C^**^	Switzerland	Case series	2	2/0	10 and 10	74%
Blondiaux et al. (37)	April 2020	Diagnosed with K.D.^*^	France	Cohort	4	1/3	9.25	6
Felstein et al. (38)	March 15–May 20, 2020	MIS-C^**^	USA	Cohort	186	115/71	8.3 (3.3–12.5)	9
Riollano-Cruz et al. (39)	April 24–June 19, 2020	MIS-C^**^	USA	Cohort	15	11/4	12.13	7
Lima-Setta et al. (40)	March—July 2020	MIS-C^**^	Brazil	Cohort	56	39/17	6.2 (2.4–10.3)	8

* *Diagnosed with KD according to the American Heart Association indications (2017) (41)*.

** *Diagnosed with KD-like children; diagnosed based on CDC recommendation (42)*.

*** *Patients are diagnosed with COVID-19 by their clinical manifestations*.

a *Obtained from Checklist from CARE guidelines for case reports (percent) (14)*.

b *Obtained from Newcastle-Ottawa Quality Assessment Form for Cohort Studies (out of nine) (15)*.

### COVID-19 Related Features

In 16 studies COVID-19 was diagnosed in all patients based on their PCR result, serological findings, or computed tomography (CT) results. Fourhundred-fifty-five patients had positive serological indices (IgG, IgA, and IgM) for SARS-CoV-2 infection, while 175 patients were tested positive for COVID-19 using PCR (Table 2).

**Table 2:** COVID-19 related features of KD-like syndrome patients, including serological, PCR, and imaging findings.

**References**	**Population**	**Type**	**Sample size**	**Positive IgG (%)**	**Positive IgA (%)**	**Positive IgM (%)**	**Positive nasopharyngeal PCR (%)**	**Positive fecal PCR (%)**	**CT-scan (%)**	**Diagnosed with COIVD-19 (%)**
Jones et al. (16)	Diagnosed with K.D. (32)^*^	Case report	1	NR	NR	NR	100	NR	NR	100
Grimaud et al. (17)	MIS-C^**^	Retrospective cohort	20	100.0		NR	50.0	10.0	5.0	100.0
Toubiana et al. (18)	MIS-C^**^	Cohort	21	90.5	NR	NR	38.1	NR	44.4	90.5
Rivera-Figueroa et al. (19)	MIS-C^**^	Case report	1	NR	NR	NR	100.0	NR	NR	100.0
Balasubramanian et al. (20)	MIS-C^**^	Case report	1	NR	NR	NR	100.0	NR	NR	100.0
Verdoni et al. (21)	Diagnosed with KD (32)^*^	Retrospective cohort	10	80.0	NR	30.0	20.0	NR	NR	80.0
Belhadjer et al. (22)	MIS-C^**^	Case Series	35	80.0	71.4	5.7	34.2	5.7	NR	88.6
Licciardi et al. (23)	MIS-C^**^	Case Series	2	100.0	NR	100.0	0.0	NR	NR	100.0
Deza Leon et al. (24)	MIS-C^**^	Case report	1	NR	NR	NR	100.0	NR	NR	100.0
Dolinger et al. (25)	MIS-C^**^	Case report	1	NR	NR	NR	100.0	NR	0.0	100.0
Riphagen et al. (26)	MIS-C^**^	Case Series	8	NR	NR	NR	0.0	NR	NR	100.0^***^
Waltuch et al. (27)	MIS-C^**^	Case Series	4	100.0	NR	NR	0.0	NR	NR	100.0
Labé et al. (28)	Diagnosed with K.D. (32)^*^	Case Series	1	NR	NR	NR	0	N.R.	1	100
Toubiana et al. (29)	Diagnosed with KD (32)^*^	Retrospective cohort	17	87.5	NR	NR	41.2	NR	NR	82.3
Chiotos et al. (30)	MIS-C^**^	Case Series	6	100.0	NR	NR	50.0	NR	NR	100.0
Rauf et al. (31)	MIS-C^**^	Case Series	1	NR	NR	NR	0	NR	NR	0.0
Pouletty et al. (32)	Diagnosed with K.D.^*^	Cohort	16	87	NR	NR	56	40	31	93.75
Dufort et al. (33)	MIS-C^**^	Cohort	99	99	NR	NR	51	NR	39	100
Capone et al. (34)	MIS-C^**^	Cohort	33	90.9	NR	NR	27.27	NR	NR	100
Whittaker et al. (35)	MIS-C^**^	Cohort	58	83	NR	NR	26	NR	NR	78
Dallan et al. (36)	MIS-C^**^	Case series	2	100	0	0	0	0	100	100
Blondiaux et al. (37)	Diagnosed with K.D.^*^	Cohort	4	100	NR	0	0	0	NR	100
Felstein et al. (38)	MIS-C^**^	Cohort	186	62	NR	NR	59	NR	NR	70.43
Riollano-Cruz et al. (39)	MIS-C^**^	Cohort	15	100	NR	NR	47	NR	73.3	100
Lima-Setta et al. (40)	MIS-C^**^	Cohort	56	61.3	NR	NR	45.2	NR	52.1	100

* *Diagnosed with KD according to the American Heart Association indications (2017) (41)*.

** *Diagnosed with KD-like children; diagnosed based on CDC recommendation (42)*.

*** *Patients are diagnosed with COVID-19 by their clinical manifestations*.

*Data are expressed as percentage of patients*.

### Laboratory Characteristics of the Patients

Twenty one studies showed the CRP level; CRP was increased in all patients who participated in all studies. Highest CRP was reported in Deza Leon et al. study (450 mg/L) (24). A higher ESR level was also reported in 9 studies. In most studies, patients had leukopenia with marked lymphopenia, hypoalbuminemia, and increased ferritin, as well as hyponatremia. Laboratory Characteristics of the patients are shown in Table 3.

**Table 3:** Laboratory findings of KD-like syndrome patients in included studies.

**Author**	**Sample size**	**References**	**CRP (mg/L)**	**ESR (mm/h)**	**Sodium (mEq/L)**	**Albumin (g/dL)**	**Ferritin (ng/mL)**	**Absolute leukocyte count (×109/L)**	**Absolute neutrophil count (×109/L)**	**Absolute lymphocyte count (×109/L)**
Jones et al.	1	(16)	133	70	133	2.8	NR	NR	NR	NR
Grimaud et al.	20	(17)	251 (94–458)	NR	131 (122–139)	2.1 (1.7–2.6)	NR	NR	10.9 (1.5–34.2)	NR
Toubiana et al.	21	(18)	253 (89–363)	NR	NR	2.1 (1.6–3.7)	NR	17.4 (5.4–42.8)	13.6 (3.3–36.4)	1.1 (0.4–5.6)
Rivera-Figueroa et al.	1	(19)	NR	72	121	2	1,030	40	NR	NR
Balasubramanian et al.	1	(20)	120	NR	133	2.6	1,496	23	20.4	NR
Verdoni et al.	10	(21)	250 (153)	72 (24)	130.8 (3.9)	3.2 (0.3)	1,176 (1,032)	10.8 (6.1)	9.1 (6.6)	0.9 (0.4)
Belhadjer et al.	35	(22)	241 (150–311)	NR	NR	NR	NR	16.0 (12.0–23.0)	13.0 (8.0–19.0)	NR
Licciardi et al.	2	(23)	NR	NR	NR	NR	58,0897	NR	NR	NR
Deza Leon et al.	1	(24)	450	56	118	2.8	699	13.3	9.8	2
Dolinger et al.	1	(25)	79.8	64	NR	2.9	2,140	NR	NR	NR
Riphagen et al.	8	(26)	301 (169–556)	NR	NR	2.2 (1.8–2.5)	602.5 (277–42,20)	NR	NR	NR
Waltuch et al.	4	(27)	267.25 (202.2–363.8)	64.5 (46–92)	NR	NR	1,023 (288–2,010)	8.25 (5.1–17)	NR	0.338 (0.25–0.61)
Labé et al.	1	(28)	195	NR	NR	NR	NR	17.4	NR	NR
Toubiana et al.	17	(29)	219 (89–363)	NR	130 (116–134)	20 (16–37)	NR	16.8 (5.4–42.8)	11 (3.3–36.4)	NR
Chiotos et al.	6	(30)	228 (83–343)	NR	130 (125–134)	3.1 (2.4–4.3)	804 (512–1,267)	NR	11.7 (9.1–16.8)	0.71 (0.17–1.20)
Rauf et al.	1	(31)	120	70	124	2.1	600	11	8.7	1.8
Pouletty et al.	16	(32)	207 (162–263)	NR	130 (127–134)	2.1(1.9–2.3)	1,067 (272–1,709)	11.5 (9–14.4)	9.2 (7.6–10.7)	1.15 (0.8–1.7)
Dufort et al.	99	(33)	219 (150–300)	61.5 (43-77.5)	NR	3.1 (2.5–3.6)	552 (305–820)	10.4 (6.7–14.5)	NR	NR
Capone et al.	33	(34)	206 (122–291)	NR	133 (131–135)	3.4 (3.0–3.7)	640 (313–1,192)	9.4 (7.19–12.33)	NR	0.8 (0.49–1.42)
Whittaker et al.	58	(35)	299 (156–338)	NR	NR	2.4 (2.1–2.7)	610 (359–1,280)	17 (12–22)	13 (10-19)	0.8 (0.5–1.5)
Dallan et al.	2	(36)	NR	NR	NR	NR	NR	NR	NR	NR
Blondiaux et al.	4	(37)	309.25	NR	130.25	NR	NR	NR	NR	0.6
Felstein et al.	186	(38)	NR	NR	NR	NR	NR	NR	NR	NR
Riollano-Cruz et al.	15	(39)	241.98	NR	NR	3.13	1470.93	NR	NR	NR
Lima-Setta et al.	56	(40)	150 (91–336)	92.5 (49.3–120.0)	NR	2.7 (2.2–3.0)	464.5 (187–852.7)	23.9 (18.35–26.00)	NR	0.796 (0.479–1.048)

*CRP, C-reactive protein; ESR, erythrocyte sedimentation rate; NR, not reported; quantitative data are expressed as mean (S.D.) or median (Range) and qualitative data are expressed as percentage of patients*.

### Patients Outcomes

Twenty-two studies reported echocardiography findings of their patients. In six studies, all patients had abnormal echocardiography. The lowest rate of abnormality in echocardiography is demonstrated in Whittaker et al. (35) study (31.03%). Slightly increased troponin level and decreased ejection fraction, as well as increased BNP on admission, were reported. In terms of respiratory outcomes, ventilation was conducted in all patients in six studies. The lowest rate of ventilation is 20 percent revealed in Feldstein et al. study (38). The most common types of ventilation are invasive, non-invasive, and nasal high oxygen flow, respectively. In 11 studies, all patients required intensive care unit admission. Pouletty et al. (32) reported the lowest rate of ICU admission. Patients outcomes and drugs used to treat patients are summarized in Table 4.

**Table 4:** Treatments and clinical outcomes of KD-like syndrome patients in included studies.

**Author**	**Sample size**	**References**	**Cardiac outcomes**				**Respiratory outcomes**				**Treatment**			
			**Abnormal echocardiography**	**Ejection fraction (%)**	**Troponin (ng/L)**	**BNP (ng/L)**	**ventilation**				**ICU Admission**	**IVIG**	**Anticoagulant drugs**	**Corticosteroid**	**Vasoactive drugs**	**Monoclonal antibodies (e.g., Infliximab)**
							**Invasive**	**Non-invasive**	**High flow oxygen**	**Total**						
Jones et al.	1	(16)	0	NR	NR	NR	N.R.	NR	N.R.	NR	0	100	100^*^	0	NR	NR
Grimaud et al.	20	(17)	NR	35 (25–55)	269 (31–4607)	3,405 (179–19,013)	40	55	5	100	100	NR	0^*^	10	100	10
Toubiana et al.	21	(18)	76	42 (10–57)	282 (10–6,900)	3,354 (16–16,017)	NR	NR	NR	52	81	100	100^*^	33	71	NR
Rivera–Figueroa et al.	1	(19)	100	NR	60	NR	NR	NR	100	100	100	100	100^*^	100	NR	0
Balasubramanian et al.	1	(20)	0	NR	NR	NR	NR	NR	100	100	100	100	100^*^	0	NR	100
Verdoni et al.	10	(21)	40	NR	1,004 (1,862)	1,255 (929)	NR	NR	NR	NR	NR	100	20^*^	80	20	NR
Belhadjer et al.	35	(22)	100	32 (9)	347 (186–1267)	57,430 (26,480 – 119,090)	62	32	NR	NR	100	71	65^**^	34	80	8
Licciardi et al.	2	(23)	100	NR	NR	NR	0	50	0	50	NR	50	0^*^	100	50	NR
Deza Leon et al.	1	(24)	100	NR	114	NR	100	0	0	100	100	100	100^*^	0	0	NR
Dolinger et al.	1	(25)	NR	NR	NR	NR	NR	NR	NR	NR	NR	100	100^***^	0	0	100
Riphagen et al.	8	(26)	87	NR	83.5 (25–813)	NR	63	25	12	100	100	100	75^*^	87	87	12
Waltuch et al.	4	(27)	75	NR	35 (10–320)	1266.5 (724–30,685)	NR	NR	NR	NR	100	75	50^***^	NR	50	100
Labé et al.	1	(28)	NR	N.R.	NR	NR	NR	NR	NR	NR	NR	NR	NR^*^	NR	NR	NR
Toubiana et al.	17	(29)	47	38 (10–57)	136 (10–6,900)	28,790 (160–160,170)	NR	NR	NR	59	76	100	100^*^	29	59	NR
Chiotos et al.	6	(30)	83	27 (19–38)	300 (50–1,390)	7,970 (5,180–186,050)	50	33.34	0	83	100	100	50^*^	100	83	NR
Rauf et al.	1	(31)	100	35	29	80,000	NR	NR	NR	N.R.	100	100	100^*^	100	100	NR
Pouletty et al.	16	(32)	69	35(32–46)	58 (36–165)	4,319 (2,747–6,493)	12.5	18.75	NR	25	43.75	93	93	25	NR	12.5
Dufort et al.	99	(33)	52	NR	NR	NR	10	NR	16	26	80	70	NR	64	62	NR
Capone et al.	33	(34)	48	NR	31 (6–78)	332.5 (64–677.6)	18	NR	NR	18	79	100	88	70	76	24
Whittaker et al.	58	(35)	31.03	NR	45(8–294)	78.8 (17,4–1054.8)	43	NR	NR	43	100	71	NR	64	NR	19
Dallan et al.	2	(36)	50	NR	NR	NR	0	100	0	100	50	NR	NR	NR	NR	NR
Blondiaux et al.	4	(37)	100	56.75	1404.25	2394.25	25	NR	NR	25	100	100	75	75	75	0
Felstein et al.	186	(38)	9	NR	NR	NR	20	NR	NR	20	80	77	47	49	48	21
Riollano–Cruz et al.	15	(39)	87	NR	2,562	143.95	20	33	NR	53	93.3	80	100	20	NR	13
Lima-Setta et al.	56	(40)	60.71	NR	200 (100–8,700)	581.8 (60.38–1274.8)	11	25	2	36	NR	89	45	55	NR	NR

* *Aspirin*,

** Heparin, and

*** *enoxaparin were used as anticoagulants*.

*N.R., not reported; ICU, intensive care unit; BNP, brain natriuretic peptide; IVIG, intravenous immunoglobulin; quantitative data are expressed as mean (S.D.) or median (Range), and qualitative data are expressed as a percentage of patients*.

### Quality Assessment

In this study, the highest and the lowest score of included case-reports/case-series based on CARE guidelines was 93 and 74%, respectively. The cohort studies included in this study were considered as high quality in seven articles and medium quality in five articles. This scale classifies papers as high quality (8–9 points), medium quality (6–7 points), and low quality (<6 points) (43).

## Discussion

KD is a vasculitis of early childhood, which is the most common reason for acquired heart disease in children in developed countries (41). Although more than 40 years of study has tried to explain it, the etiology of KD remains unidentified (44). So far, researches have been able to determine immune response to a stimulus as a significant factor in the pathogenesis of the disease; however, researchers have not been able to identify the stimulus yet (45, 46). One probable explanation is exposure to an infectious agent and subsequently triggering the immune system. This idea can explain the peak of KD cases in winter (47, 48).

In the Dean et al. study in a KD epidemic in Hawaii, 44 percent of the cases had a history of respiratory infection in the month before their visit. In their research, although an infectious agent is proved as one of the immune system triggers and an etiology for KD, yet the analysis failed to determine the microorganism responsible for it (49). Other studies suggested viral agents such as adenoviruses as possible etiologies (50–52).

Members of the coronaviridae family have also been suggested as triggers for KD (53). Some studies before the COVID-19 pandemic showed that 7 percent of the patients with KD symptoms had positive PCR for at least one of the coronaviridae family members (54). Among the family, the new Haven coronavirus (HCoV-NH), which is similar to HCoV NL-63, has drawn more attention (55–57). Esper et al. revealed in their study that 72.7 percent of KD patients tested positive for HCoV-NH by PCR (58). Even though other studies did not fully support Esper's findings (55, 59), yet it pointed out that the studies that searched for the virus traces via serological tests showed a higher rate of virus detection than the studies that utilized PCR (60).

With the incidence of the COVID-19 pandemic, the number of children who presented with KD symptoms increased dramatically. The Verdoni et al. study showed that the monthly incidence of KD in an Italian province had increased 30-folds (21). The significant inflammatory response of the body to the novel coronavirus alongside the epidemiological studies have been in favor of the theory that suggests COVID-19 as a trigger for the immune system and an etiology for KD's (61–63).

This review has gathered the findings of 25 articles that have presented patients with KD-like syndrome in the era of the COVID-19 pandemic. Most of the patients have exhibited traces of COVID-19 in their tests. Among these data, it seems that KD-like syndrome are more strongly associated with positive serology tests. To justify this finding, we can point out that PCR turns negative in a shorter period compared to serology (measuring immunoglobulin levels) tests (64). It seems that the immune response that will lead to KD-like syndrome needs an amount of time to develop. This time exceeds the time in which the PCR test will result positive. We should note that a small fraction of the patients with KD-like syndrome did not reveal any trace of the novel coronavirus in their tests. This result could be due to the limited sensitivity of serology and PCR tests (65–67), or it could simply be due to the normal incidence of KD because of other etiologies. The current study suggests that in these days of the pandemic, it is highly recommended that children who present with KD or KD-like syndrome in the regions affected with COVID-19, be tested for SARS-CoV-2 infection. It seems that serology tests are preferred to PCR; however, we recommend that both tests be conducted for the patients.

In most reviewed articles, leukopenia and especially lymphopenia decreased levels of serum albumin, and an increase in Ferritin, ESR, and CRP was noted. Impaired heart function and myocarditis are the unfortunate outcomes of KD-like syndrome (68, 69). The articles have measured troponin and BNP levels and also conducted echocardiography for patients. Despite having near-normal levels of troponin, most children had increased BNP and decreased ejection fraction that confirmed the previous studies on KD. It seems that the risk of developing heart complications is increased when KD symptoms has co-occurred with COVID-19 compared to when the sole problem is KD. Based on the studies, we strongly recommend heart function screening for patients who are suspected of having KD-like syndrome.

KD is rare disease, which was mainly managed by pediatric rheumatologists, but as a result of COVID-19 the prevalence of this disease and KD-like syndrome has increased notably and these disease spectrums are now considered as a health issue. Although most of the articles reviewed in this study have similar findings, this systematic review assists clinicians to determine the best therapeutic approach for their patients based on their demographic, clinical, and laboratory findings to achieve the best outcome.

Although the clinical findings of KD is very similar to KD-like syndrome, there are some differences including the older age of KD-like Syndrome patients, which could be justified due to the higher exposure of school-aged children and adolescents to the virus compared to infants and toddlers. In addition, gastrointestinal Symptoms, lymphopenia, thrombocytopenia, and hypertriglyceridemia are more prevalent in KD-like syndrome compared to KD. KD-like syndrome patients are at higher risk of complications compared to KD patients. Moreover, the inflammatory factors have had more noticeable changes in these new patients, which may be related to primary infection with SARS-CoV-2 rather than KD itself (70, 71) (Table 5).

**Table 5:** The comparison of KD and KD-like syndrome.

	**KD**	**KD-like syndrome**	**Common in both KD and KD like syndrome**
Clinical findings	More prevalent in infants and toddlers	More prevalent in adolescents and older children, GI symptoms and heart failure are more common	Prolonged fever, fissured lips, Non-exudative conjunctivitis, and hypotension
Laboratory findings	Lower rate of elevated ferritin	Thrombocytopenia, Lymphopenia, Hypertriglyceridemia higher rate of elevated ferritin	Neutrophilia, increased CRP
Outcome	Better prognosis and lower rate of ICU admission. Coronary artery changes and rarely with decreased of ventricular function are common	Poorer prognosis, higher rate of ICU admission. Faster advancement of symptoms. Ventricular dysfunction, coronary artery changes, atrioventricular valve regurgitation and pericardial effusions are common	

### Limitations

It should be noted that there are some diagnostic limitations due to the relatively low sensitivity of serological test compared to PCR. Moreover, most of the articles that were reviewed in this study were case reports and had relatively low evidence. We found only a few articles concerning our subject (Even with lower evidence level). It is necessary to perform pathophysiological assessments in addition to conducting case-control studies so that we would be able to understand the relationship between the novel coronavirus and KD better.

## Conclusion

Incidence of children presenting with a severe inflammatory syndrome with KD-like features are increased during the COVID-19 pandemic. These children present with more severe symptoms which is attributed to worse clinical outcome and require intensive treatment and close monitoring. This study indicates that serological indices for SARS-CoV-2 have stronger correlation with the KD-like disease incidence compared to PCR. Moreover, CRP could also be helpful in terms of diagnosis. Although only one patient in included studies died of the complications the disease, most of the patients required ICU admission.

## Data Availability Statement

The raw data supporting the conclusions of this article will be made available by the authors, without undue reservation.

## Author Contributions

MQ and KP: conceptualization. ME and MQ: search. PM and MA: data extraction. PM, ME, and MQ: writing—original draft. PI and KP: writing—review & editing. All authors read and approved final version of the manuscript.

## Conflict of Interest

The authors declare that the research was conducted in the absence of any commercial or financial relationships that could be construed as a potential conflict of interest.

## Abbreviations

COVID-19: coronavirus disease 2019
KD: Kawasaki disease
SARS-CoV-2: Severe acute respiratory syndrome coronavirus 2
MIS-C: Multisystem inflammatory syndrome in children
CARE: guidelines for Case Reports
NOS: The Newcastle-Ottawa Scale
ICU: intensive care unit
CRP: C-reactive protein
ESR: erythrocyte sedimentation rate
N.R.: not reported
BNP: brain natriuretic peptide
IVIG: intravenous immunoglobulin.
