# Supplementary material for: Characteristics of Children With Kawasaki Disease-Like Signs in COVID-19 Pandemic: A Systematic Review
Source: Front Pediatr. 2021 Mar 18;9:625377. doi: 10.3389/fped.2021.625377 (PMC8012548; doi:10.3389/fped.2021.625377)
Supplement: Supplementary file 1 [file Table_1.DOCX]

**Supplementary table**

| 1. | "covid 19" OR "covid-19" OR "*covid-19*" OR "*covid*"OR "*SARS-CoV-2*"OR "*2019-nCoV*"OR "*novel coronavirus*"OR "*new coronavirus*"OR "*coronavirus*" |
| --- | --- |
| 2. | Kawasaki OR KD OR Kawasaki-like OR multi-system OR multi-system) AND inflammatory AND (syndrome* OR disease)) OR MISC OR MIS-COR PIMS OR (inflammatory AND (multi-system OR multi-system) AND (syndrome*OR disease*)) OR Systemic Inflammatory Response Syndrome OR hyperinflammatory |
| 3. | child* OR pediatric |
| 4. | #1 AND #2 AND #3 |
| Filters | English; Publication date in the last one year (Done on July 1, 2020); |
